# Scrutinizing Stator Rotation in the Bacterial Flagellum: Reconciling Experiments and Switching Models

**DOI:** 10.3390/biom15030355

**Published:** 2025-03-01

**Authors:** Ayush Joshi, Pushkar P. Lele

**Affiliations:** 1 Artie McFerrin Department of Chemical Engineering, Texas A&M University, College Station, TX 77480, USA; ayushjoshi2911@tamu.edu; 2 Department of Biomedical Engineering, Texas A&M University, College Station, TX 77840, USA

**Keywords:** FliG, proton-motive force, peptidoglycan, ATP synthase, mechanosensor

## Abstract

The bacterial flagellar motor is one of the few known rotary motors, powering motility and chemotaxis. The mechanisms underlying its rotation and the switching of its rotational direction are fundamental problems in biology that are of significant interest. Recent high-resolution studies of the flagellar motor have transformed our understanding of the motor, revealing a novel gear mechanism where a membranous pentamer of MotA proteins rotates around a cell wall-anchored dimer of MotB proteins to turn the contacting flagellar rotor. A derivative model suggests that significant changes in rotor diameter occur during switching, enabling each MotA_5_MotB_2_ stator unit to shift between internal and external gear configurations, causing clockwise (CW) and counterclockwise (CCW) motor rotation, respectively. However, recent structural work favors a mechanism where the stator units dynamically swing back and forth between the two gear configurations without significant changes in rotor diameter. Given the intricate link between the switching model and the gear mechanism for flagellar rotation, a critical evaluation of the underlying assumptions is crucial for refining switching models. This review scrutinizes key assumptions within prevailing models of flagellar rotation and switching, identifies knowledge gaps, and proposes avenues for future biophysical tests.

## 1. Introduction

The bacterial flagellar motor (BFM) is an extraordinary nanomachine that rotates the extracellular flagellar filament to enable motility and chemotaxis in many bacterial species [1,2,3]. Among the few rotary motors in biology [4], the BFM has been the subject of extensive research over the past decades. The flagellar motor comprises a rotor connected to the extracellular filament and a stator complex that delivers torque to rotate the flagellum [5]. The stator complex consists of multiple independent units that utilize ion or proton flux across the membrane to function [6,7,8,9,10]. Other than rotating the flagellum, the stator also functions as a mechanosensor: it senses sudden changes in the external viscous resistance to flagellar rotation and dynamically remodels the number of stator units engaged with the rotor [11]. The mechanosensitive flagellar stator is likely involved in downstream signaling responsible for biofilm formation, genetic competence, and pathogenesis [12,13,14].

In *Escherichia coli*, each stator unit is made up of MotA and MotB proteins [15]. The MotB subunits anchor within the peptidoglycan layer, with the membranous MotA subunits contacting the rotor [16]. Stator units harness the free energy of proton translocation down the electrochemical gradient to drive motor rotation. This is achieved by coupling proton binding/unbinding at key residues to the relative motion of MotA and MotB. Strong electrostatic interactions between charged residues on MotA and its rotor counterpart, FliG, facilitate the transmission of torque to the rotor [17,18,19].

**Figure 1:**
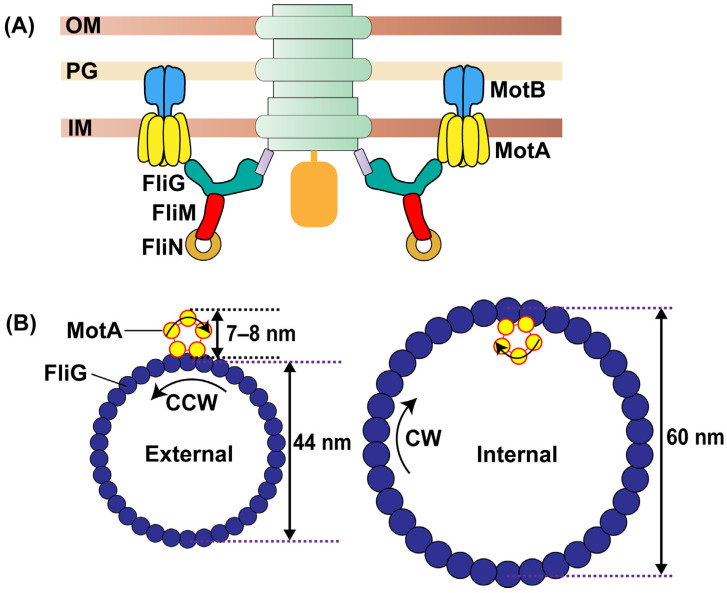
(**A**) The flagellar motor. The C-ring consisting of FliG, FliM, and FliN complexes is indicated by teal, red, and orange colors. Independent stator units consisting of MotB and MotA subunits are indicated in blue and yellow, with MotB anchoring to the cell wall. (**B**) Derivative Switching Model. The MotA pentamer (small gear, yellow) rotates CW-only, causing the FliG ring (blue) to rotate CCW in the external gear configuration. The FliG ring is predicted to undergo large conformational changes, expanding to engage with the distal interface of the MotA pentamer—see Figure 4 in ref. [41] and Figure 5 in ref. [44]. This causes the motor to rotate CW in the internal gear configuration. In the hypothetic scenario where these complexes are rigid and lack flexible domains in the two states (CW and CCW), the predicted expansion must be by a distance equal to the separation between the proximal and distal residues of MotA that interact with the FliG torque helix. This equals ~14–16 nm. Such a large hypothesized change in FliG diameter has not been observed experimentally [46].

The flagellar rotor contains several components [20], but three conserved proteins are crucial for core architecture and interactions with the stator: FliG, FliM, and FliN [21]. Together, these three complexes form the cytoplasmic ring, or simply the C-ring (Figure 1A). MotA delivers torque to a ring of FliG consisting of 34 subunits [22,23], rotating the entire flagellar assembly, including the C-/MS-ring, flagellar rod, and the extracellular flagellar hook and filament [24]. The rotation is further stabilized by the peptidoglycan (P) and lipid (L) rings that surround the flagellar rod [23,25,26,27,28,29,30]. Similarly to stator units, the FliM and FliN complexes exhibit dynamic remodeling, with biophysical studies indicating variable subunit stoichiometry in response to environmental stimuli [31,32,33]. However, structural evidence is mixed, with some studies reporting variable C-ring symmetry and others reporting fixed stoichiometry [23,34,35]. Continuing advances in cryo-EM approaches may likely resolve these differences, revealing the actual dynamic picture of motor complexes, including that of the *E. coli* stators that have thus far been challenging to visualize with this technique.

The C-ring lies at the heart of chemotaxis in motile bacterial species [36]. It helps the motor switch its direction of rotation between counterclockwise (CCW) and clockwise (CW) in many species. Cells of *E. coli* and *Salmonella enterica* carry multiple flagella that bundle together when they rotate CCW in unison. This propels the cell in a straight ‘run’ [37,38]. A switch from the default CCW to the CW direction in one or more of the flagella causes the cell to ‘tumble’, and it generally picks a new direction when CCW rotation resumes [39,40]. The cell modulates reversals between these two rotational directions to navigate toward favorable chemical habitats. The mechanism of flagellar switching is of considerable scientific curiosity and significance [41].

Exciting advances in high-resolution studies of the flagellar stator have transformed our understanding, offering new insights that may bridge the gaps in our knowledge of flagellar motor rotation and switching [42,43]. The latest model of flagellar rotation suggests that the stator itself is a rotary device, with multiple MotA subunits functioning as a tiny ‘rotor’ rotating around a MotB dimer anchored to the cell wall. The stator could be viewed as a small gear that turns the larger gear (FliG ring). This stator rotation model is intricately linked to the mechanism of switching in the direction of rotation [44]. According to a derivative switching model, large changes in the size of the FliG ring alter the stator–rotor interactions, positioning the stator between internal and external gear configurations (Figure 1B). This induces switches between CCW and CW directions of rotation [41,42,43,45].

In this perspective, we highlight intriguing inconsistencies between the derivative switching model and new structural data, indicating a more complex mechanism than initially anticipated. We propose directions for future investigations to fill the remaining gaps in our understanding and critically assess revised models of motor function.

## 2. Stator Structure and Operation

The latest structural data indicate that each stator unit is made up of a dimer of MotB and a pentamer of MotA [42,43]. MotB contains a C-terminal domain that anchors within the peptidoglycan [47], referred to hereafter as the peptidoglycan-binding domain (PGBD), and a transmembrane helix (TMH), with the N-terminal domain contained within the membrane. MotA has multiple TMHs and a cytoplasmic loop containing charged residues crucial for engaging with FliG in *E. coli* [48]. Two of the four TMHs in each MotA run parallel to MotB’s membrane helices, with the MotB regiocarrns fitting snuggly within a pentagonal cylinder comprising these MotA TMHs. Critical to stator function is a conserved MotB residue, Asp32 (D32), that protonates and deprotonates to shuttle the proton from the periplasm to the cytoplasm [47,49].

A maximum of ~11–12 stator units can surround the *E. coli* rotor [50,51]. The cell optimizes stator engagement, perhaps because the proton flux tends to be high when the viscous resistance (viscous load) to flagellar rotation is minimal, and the motor rotates at top speeds [52]. This can occur when the motor is newly assembled, and the nascent filament is still growing [53]. High proton flux could short-circuit the membrane, inhibiting cell growth. Consistent with this possibility, *E. coli* cells appear to engage no more than one stator unit at low loads to rotate the flagellum [11].

The stator complex in *E. coli* can remodel in response to changes in the load because its stator units can reversibly detach from the motor, with unbinding rates decreasing exponentially as the torque applied to the rotor increases. At low (high) loads, the torque delivered by the stator unit is low (high), resulting in fewer (greater) engaged stator units [54,55]. This load-dependent remodeling of the stator complex might arise from the presence of a catch-bond [56]. However, a catch-bond is not necessary; an alternative model suggests that higher viscous loads increase the reaction torque acting at the MotB-peptidoglycan interface, causing the so-called PGBD to entangle locally within the polymeric cell wall. As a result, the residence time of the stator unit at the motor increases [53,57]. Irrespective of the mechanism, the rotational rates tend to be low even when numerous stator units are engaged at high loads. As a result, the extracellular load itself can be viewed as an effective inhibitor of proton flux through engaged stator units [13].

In species with larger rotors, as many as 16 stator units can be observed to be associated with the motors [58,59]. There is no evidence yet that these motors dynamically remodel their stator complex. Notably, in species such as *H. pylori*, the cell expends considerable resources in assembling what resembles a locking cage around the stator complexes [60,61]. Although the cage’s functional role remains unclear, it likely inhibits the exchange of stator units with the pool of units in the membrane. Perhaps, proton leakage through motor-bound stator units in *H. pylori* may be insufficient to significantly impact cell growth, unlike in *E. coli*. Another possibility is that *H. pylori*’s slow doubling rate and frequent exposure to acidic conditions in the stomach desensitizes it to proton leakage through motor-bound stator units.

Nevertheless, there are ~ 100–200 stator units in the cell [62], and most do not associate with the motors at a given instant. Presumably, the cell prevents proton leakage through these diffusive stator units with a plug region encoded by residues 51–70 in MotB [63]. The two plugs in the MotB dimer operate in *trans* to block the passage of protons until the stator unit interacts with FliG. At that instant, the plugs open, setting the stage for its proton-dependent operation. The plugs are highly effective, with minimal proton leakage even when stator proteins are overexpressed [64]. Deleting the MotB plug region, termed ‘plugless’ MotB hereafter, and overexpressing the mutant with MotA leads to high proton leakage, impairing cell growth [63,64]. Interestingly, motility remains intact. The plug region is conserved across different species, underscoring its importance in preventing cytoplasmic acidification by disengaged stators.

The opening of the plugs is not the only required condition for torque to be delivered to the rotor. To achieve torque transmission, the so-called PGBD must be first anchored in the peptidoglycan layer. The PGBD is in a retracted position in its usual state, enabling the stator units to diffuse within the membrane. When MotA interacts with FliG, the PGBD extends and attaches to the cell wall along with the opening of the plug [65]. The mechanism by which FliG induces these conformational changes in MotB remains unclear at this time. However, it is also likely to be influenced by mechanical tug and pull between FliG and MotA [66].

## 3. Evidence for Stator Rotation and the Derivative Switching Model

High-resolution cryo-EM studies of purified stator complexes have enabled atomic models of the pentameric MotA surrounding the N-terminal transmembrane helices of MotB [42,43]. Studies of different types of purified *Campylobacter jejuni* stator complexes—wild-type MotA_5_MotB_2_, MotA_5_MotB_2_^plugless^, and MotA_5_MotB_2_^plugless-D22N^ (D22N in *C. jejuni* and D32N in *E. coli*)—all appeared similar. No evidence was found for a previously predicted large conformational change [47,67,68,69]. The so-called proton half-channels through which the proton passes from the periplasm into the cytoplasm were not readily observed in the plugged MotA_5_MotB_2_ units, although they could be discerned in the MotA_5_MotB_2_^plugless^ units. The D22 pointed toward periplasm, presumably in a proton-accepting state, and the N22 pointed toward the cytoplasm, presumably mimicking a proton-releasing state.

To interpret these results, one must make a reasonable but important assumption that the coupling between proton transport and the relative movement between MotA and MotB is independent of the state of the PGBD in the MotA_5_MotB_2_^plugless^. Considering the lack of substantial conformational changes observed between protonated and unprotonated states of MotA_5_MotB_2_^plugless^ units, coupled with the 5:2 stoichiometry of the complex, it is logical to postulate that the MotA pentamer undergoes rotational movement around the MotB dimer.

A turnstile-type mechanism seems most likely, involving rotationally offset half-channels at the MotA-MotB intefaces [70]. One type of half-channel links the proton-binding sites to the periplasm, while the other links them to the cytoplasm. When the plugs are open, a proton can access one of the two critical MotB residues via the periplasmic half-channel. Protonation enables relative movement between otherwise incompatible MotA and MotB interfaces, leading to a 36-degree rotation of the pentamer around the dimer. Then, a different proton is released into the cytoplasm through a cytoplasmic half-channel. The rotational offset between the half-channels prevents proton leakage uncoupled with rotation. A full rotation of the pentamer probably requires the passage of about 10 protons. See ref [70] for a detailed discussion of the turnstile mechanism. The stator rotation model is consistent with the high-duty nature of the processive flagellar motor [71]. Further, it readily explains how reversing the direction of proton flux inverts the direction of rotation [72].

Based on the arrangement of charged residues along the inner MotA-MotB interfaces, it has been hypothesized that the pentamer rotates CW unidirectionally around the dimer [43]. This compelling idea suggests a plausible mechanism for the switch in the direction of rotation [45]. The rotating pentamer could change the direction of rotation of the FliG ring by functioning as an external driver gear during CCW motor rotation and as an internal driver gear during CW motor rotation [41,43,45,73].

**Figure 2:**
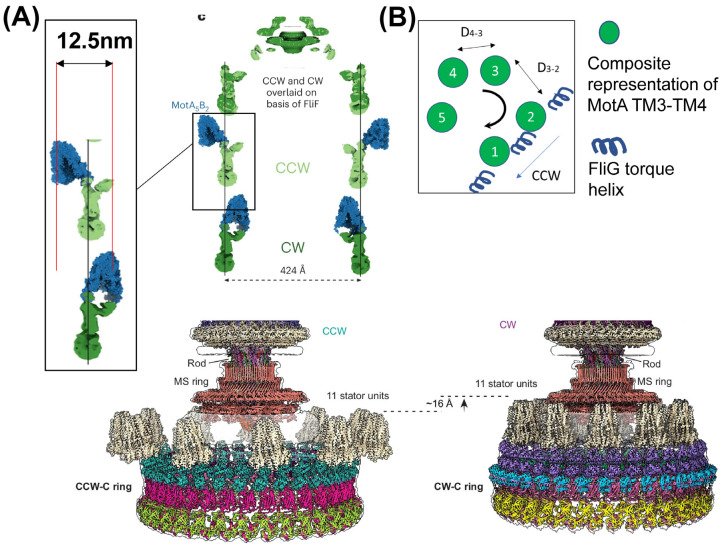
(**A**) Revised switching model. Recent works suggest a model in which the overall dimensions of the FliG ring remain almost unchanged during switching but that the stator units move closer to the basal body in the CW state [75,76]. Pseudo-atomic models depict ~12 nm displacement of the top of the MotA pentamer between the CW and CCW states [75,76]. Reproduced with permission from Springer Nature. (**B**) Imperfections in MotA-FliG arclengths: MotA–MotA spacing is variable within pentamers that are asymmetric [42]: i.e., D_4–3_ ≠ D_3–2_. These and other factors can contribute to unequal traversing of arclength during the 72-deg rotational displacement of the MotA subunits. However, flexibility within the torque-helix/FliG domains and in the MotA–MotA spacing could help deform the interfaces, compensating for minor imperfections.

Past studies have schematically depicted this attractive hypothesis regarding the switch in the direction of flagellar rotation by indicating an expansion of the FliG ring [41,43,44,74]. The proposed large expansion in FliG diameter brings the distal side of the MotA pentamer in contact with FliG to promote CW rotation, while the proximal side of the MotA pentamer contacts FliG during CCW rotation (see schematics in Figure 4 in ref. [41] and Figure 3 in ref. [74]). Significantly, structural data appear to suggest that the average location of the MotA residues that interact with FliG is mostly along the periphery of the ~7–8 nm cytoplasmic interface of the MotA pentamer [42]. Assuming perfectly rigid rotor and stator structures, a directional switch would necessitate a 14–16 nm FliG diameter expansion, equivalent to twice the stator diameter (Figure 1B). Such an expansion has not been observed experimentally. Instead, stator anchoring within the peptidoglycan layer likely allows for some flexibility that might enable the stator unit to swing across FliG (Figure 2A).

In the next section, we assess the major assumptions underlying stator rotation/switching models, the constraints on stator displacements, and highlight the sometimes-overlooked role of protein flexibility in determining model validity.

## 4. Assumptions in the Stator Function Models and Future Directions

### 4.1. MotB Plugs and the PGBD

Conformational information from purified structures lack the physiological context of the native cellular environment. Hence, it is helpful to first consider the activity state of plugless MotB-MotA stators within the context of the living cell. When the *E. coli* and *Salmonella enterica* plugless MotB-MotA are overexpressed in the respective hosts, the proton leakage is adequate to slow cell growth [64]. In contrast, deleting a larger region in MotB (51–100), termed simply, MotB_∆L_, which encompasses the plug region, does not inhibit cell growth at all [65,77], even though the plug is missing. A single residue change (L119P or L119E) in the MotB_∆L_ is needed to reintroduce the growth defects [64,65].

In vitro work suggests that the MotB periplasmic C-terminal fragment (MotBC2, residues 99–276) with the L119P mutation interacts with peptidoglycan extracts stronger than wild-type MotBC2 fragment. According to the authors, the L119P mutation enables better anchoring of the PGBD in the MotB_∆L_ [65]. The L119 residue lies within α1 helix of MotB, which connects the PGB core with the dispensable MotB plug region and is probably involved in PGBD extension [78]. Evidently, plug deletions do not guarantee accessibility of the proton channel. Whereas, PGBD extension may be crucial for maintaining open channels, in the wild-type and in the plugless stator mutants.

Santiveri and co-workers used a plugless MotB strain in *C. jejuni* (∆41–60), which likely leaks protons much like the corresponding plugless mutant in *E. coli*. Rigorous biophysical characterization of that mutant is awaited. A good initial step would be to investigate the role of PGBD extension in the relative movement between MotA-MotB subunits and its impact on proton transport in a model organism. Delineating the precise molecular mechanisms by which MotA–FliG interactions trigger PGBD extension will provide critical insights and reinforce the rigor of the stator rotation model.

### 4.2. FliG Ring Expansion

The chemotaxis response regulator, CheY, binds to the FliM and FliN complexes when phosphorylated, promoting a change in the FliG ring from CCW to CW conformations [41,44,74,79]. The affinity of CheY-P for the motor increases with increasing torque delivered by the stator units [57]. Thus, the mechanical torque generated by stator units not only influences the PGBD state and its interactions with the cell wall but also exerts an allosteric effect on the C-ring interfaces [57]. The CheY-induced conformational changes in the C-ring ultimately alter the FliG track along which the stator units deliver torque [80,81]. Specifically, the FliG torque helix reorients by ~180 degrees relative to the MotA interface during the switch’s conformational changes [22,82].

Intriguingly, the torque helix reorientation is not accompanied by a 14–16 nm expansion of the FliG ring diameter during CW rotation. The maximum measured difference between CW and CCW FliG diameter is ~7 nm in *B. burgdorferi*. In some species, the FliG ring contracts by ~2 nm in the CW state, rather than expanding [41,45,75,76]. When FliG is fused with FliF, a part of the MS ring, the resultant motors in *S. enterica* are functional, but the C-ring diameter appears to contract by ~5–6 nm. Perplexingly, these mutant motors tend to rotate more CW than the corresponding wild-type with larger C-rings [42]. In this particular mutant, the space at the top of the C-ring appears quite limited, raising the possibility that in the internal gear configuration, the rotation of the MotA pentamer could be sterically hindered by its increased proximity to the MS ring. How the motor rotates CW in this mutant without significant FliG diameter expansion is a bit of a mystery. Detailed biophysical and structural characterization of this mutant could reveal critical insights.

If the FliG diameter does not change much in the CW and CCW states, how might the distal interface of the pentameric MotA interact with the FliG torque helix to generate torque in the CW direction? There are multiple possible explanations. First, the structures involved are flexible, with parts of FliG and MotA likely deviating by ~1–2 nm from the averaged positions reported in structural data during motor operation [82]. This flexibility likely compensates for potential mismatches between the arc length traversed by a MotA monomer during a 72-degree rotational step and the corresponding displacement the FliG torque helix undergoes, given the 34-fold C-ring symmetry. Flexibility in the interacting domains might also compensate for differences in MotA–MotA spacing in the asymmetric MotA pentamers, helping to maintain the registry between MotA and FliG subunits (Figure 2B). Nevertheless, the gap that the FliG torque helix must cover to engage with the proximal or distal interfaces of the MotA pentamer is considerable (Figure 1B). Proteins that transmit or operate under force must maintain a certain degree of rigidity; excessive flexibility can compromise force (torque) transmission, leading to motor failure under high loads. Thus, it is unclear whether domain flexibility alone is sufficient to bridge the distance between the distal and proximal MotA interfaces during the directional switch. A likelier explanation is that the stator unit undergoes lateral movement along the C-ring interface, repositioning back and forth between the internal and external gear configuration (Figure 2A).

The latest structural evidence appears consistent with the stator repositioning between the internal and external gear configurations. Johnson and co-workers remark that while the overall dimensions of the C-ring remain equivalent between states, the positioning of the stator units is vastly different [75]. Pseudo-atomic models depict a ~10–12 nm inward movement of the MotA pentamer during CW rotation [75,76] (Figure 2A). As a typical switch in the direction of rotation occurs within 1–10 ms [83], there is insufficient time for the PGBD to unanchor from the cell wall, diffuse ~ 10–12 nm relative to the rotor, and re-anchor in the cell wall. Also, frequent stator detachment from the cell wall would be inconsistent with measurements indicating that a switching motor rotates at stable speeds in either direction. It appears that the MotB linker that connects the PGBD and transmembrane domain must swing back and forth, enabling the MotA pentamer to translocate considerable distances during switches.

**Figure 3:**
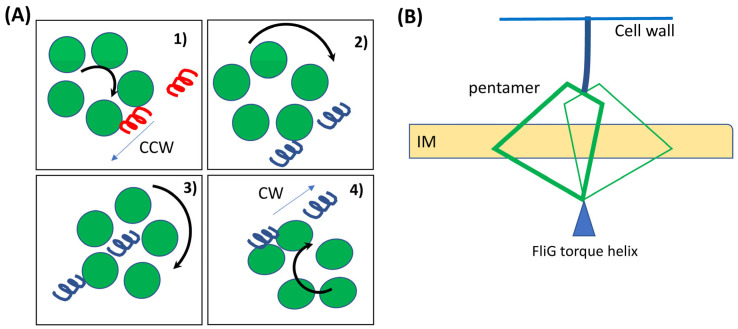
(**A**) Proposed pivoting mechanism for stator repositioning during a switch. In chronological order: (1) CW rotating pentamer causes the FliG ring to rotate CCW when the torque helix is in its default state (red). (2–3) A 180-degree reorientation of the helix (blue) causes the CW rotating pentamer to pivot about FliG by utilizing the proton-flux. (4) CW rotation of the FliG ring commences when the repositioned stator unit re-engages with the torque helix. (**B**) Issues with pentameric repositioning. If the MotB linker’s displacement is restricted by FliL or other proteins, the MotA pentamer must tilt to enable switching, irrespective of whether the pivoting mechanism is valid. Given the fixed position of the FliG torque helix in both CW and CCW states, and considering the pentamer’s dimensions (height ~9 nm, cytoplasmic diameter ~7–8 nm) along with the inner membrane’s location roughly midway through the pentamer, it is highly probable that the cytoplasmic loops of the distal MotA subunits could penetrate into the membrane in tilted stator units.

What force or torque causes the MotB linker to swing or flex perfectly in sync with erratically re-orienting FliG torque helix? One possibility is that the proton flux itself powers stator repositioning: altered electrostatic interactions between the stator and rotor during the reorientation of the FliG torque helix could cause the CW-rotating pentamer to expend several protons pivoting ~180 degrees around a point in space as shown in Figure 3A. This mechanism could explain the physical basis for the proposed role of the proton-flux in switching [84], so long as MotA remains in contact with FliG when pivoting.

Additional flagellar motor-associated proteins, such as FliL, introduce further complexity to switching in some species as they appear to fit snuggly around the periplasmic MotB domains. Although the absence of FliL does not appear to impact stator recruitment or switching in *E. coli* [54], in *B. burgdorferi* and other species, it is essential for motor function [85,86,87]. If FliL sterically limits the movement of the MotB linker to a narrow range, how might the motor switch?

One possibility is that the MotB linker remains in place, but the MotA pentamer tilts relative to the membrane (Figure 3B). In such an orientation, the pentamer requires less lateral space to act on the rotor in the internal gear configuration, potentially resolving the spatial constraints observed in FliF-G fusion experiments. However, there are limits to such tilts. For the known height and dimensions of the cytoplasmic face of the pentamer and the position of the membrane relative to the pentamer, geometric considerations suggest that the sides of the pentamer distal from the torque helix could end up inside the membrane, at least for the scenario depicted in Figure 3B. Such movement could be resisted by two out of the four MotA TMHs (TM1 and TM2) that strongly interact with the hydrophobic inner regions of the membrane. Additionally, two amphipathic MotA helices align with the upper and lower parts of the membrane [43]. These will likely provide additional resistance to pentamer tilts.

A combination of stator–rotor interface deformability, MotB linker flexibility, and restricted pentamer tilts may collectively resolve the spatial mismatches described in the scenarios above. Carefully designed biophysical and structural experiments could help test this notion and evaluate the feasibility of the different switching models in the future.

## 5. Future Directions

Previous models posited a mechanism for flagellar stator function involving substantial rearrangement within the MotA cytoplasmic domain relative to MotB. However, recent high-resolution structural data revealed a pentameric MotA ring surrounding the MotB dimer, challenging previous models and giving rise to stator rotation and switching models. While the stator rotation model offers a compelling framework for understanding the mechanisms of directional reversals of the flagellar motor, the existing structural data appear inconsistent with the derivative switching model. Revised switching models help resolve these inconsistencies but also raise new questions about the biomechanical properties of the interacting motor domains. Rigorous biophysical characterization will be essential for validating current switching models and testing their underlying assumptions.

## Data Availability

No new data were created or analyzed in this study.
